# Beyond Postpartum Fever: Case Report Uncovering Deep Septic Pelvic Thrombophlebitis

**DOI:** 10.7759/cureus.43034

**Published:** 2023-08-06

**Authors:** Autumn V Barnes, Raihan Noman, Samir Shakfeh

**Affiliations:** 1 Family Medicine, HCA Healthcare/USF Morsani College of Medicine GME: Oak Hill Hospital, Brooksville, USA; 2 Medical School, HCA Healthcare/USF Morsani College of Medicine GME: Oak Hill Hospital, Brooksville, USA; 3 Obstetrics and Gynecology, HCA Healthcare: Oak Hill Hospital, Brooksville, USA

**Keywords:** hyper coagulopathy, post partum, dspt, post-partum fever, obstetrics & gynecology

## Abstract

Deep septic pelvic thrombophlebitis (DSPT) is a rare postpartum condition that should be considered in the setting of postpartum fever and may prove to be a challenging diagnosis. Here, we report the case of a 26-year-old female who presented with fever and antibiotic-resistant leukocytosis following an uncomplicated cesarean delivery. After ruling out pulmonary embolism and other causes of septicemia and considering the overall negative imaging studies, the patient received a clinical diagnosis of DSPT and recovered well following antibiotic augmentation and anticoagulation.

## Introduction

Deep septic pelvic thrombophlebitis (DSPT) is an uncommon postpartum presentation affecting 1 in 3000 deliveries [1-2]. It is a potentially deadly subset of septic pelvic thrombophlebitis (SPT) which remains difficult and often misdiagnosed [1]. DSPT is a diagnosis of exclusion and should be considered in the setting of antibiotic-resistant fever 4 hours following delivery in postpartum women [1-3]. Here, we present a case of successful diagnosis of DSPT in a 26-year-old primigravid who presented with fever and antibiotic-resistant leukocytosis following an uncomplicated cesarean delivery. Though data is limited regarding this diagnosis, our case appears to align with others in that the patient appeared non-toxic with negative blood cultures throughout the admission and shared cesarean section as a risk factor. Our case is unique from many of those in the literature as there was no confirmed presence of septic emboli complicating the presentation. This case report was previously presented at the 2023 FAFP Spring Forum on April 16, 2023.

## Case presentation

**Figure 1:**
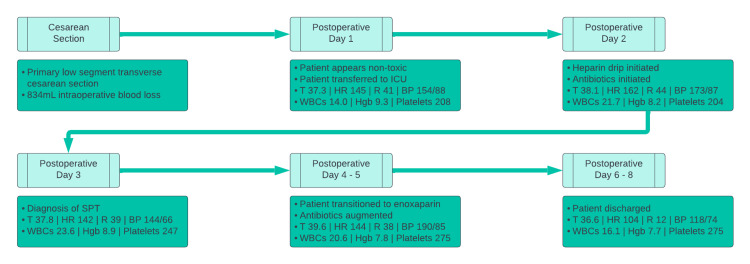
Timeline of events and findings. WBC: white blood cell; SPT: septic pelvic thrombophlebitis.

This case involves a 26-year-old primigravid woman with a past medical history of obesity and asthma, a pregnancy complicated by marijuana use and abnormal glucose in the first trimester, and a 100 lb weight gain throughout the pregnancy, who presented for elective labor induction at 40 weeks gestation. Despite adequate contractions, the patient ultimately required a cesarean section at 40 weeks and 3 days following a failure to progress beyond 5 cm dilation. A primary low-segment transverse cesarean section was performed with an estimated 834 mL of blood loss, for which the patient received 2 units of packed red blood cells. The patient’s overall hospital course timeline is depicted below in Figure 1.

The patient and infant tolerated the initial procedure well and appeared healthy following the cesarean section. On postoperative day (POD) 1, the patient felt well overall and appeared non-toxic. A physical exam later in the day revealed a tachycardic patient with a blood pressure of 154/88 and a temperature of 37.2°C. The WBC count was elevated at 14.0 x 10^9^/L. The hemoglobin level was 9.3 g/L, and the platelet count was 208 x 10^9^/L. At this time, the patient had a blood oxygen saturation of 94% O2 on room air and D-dimer was elevated to 1289 mg/L fibrinogen equivalent unit (FEU). Multiple imaging studies were performed, as outlined below. Two electrocardiograms were performed, each unremarkable except for sinus tachycardia. An echocardiogram revealed normal systolic function with an ejection fraction of 55%. Chest x-ray was negative for acute cardiopulmonary disease. An initial CT angiography with contrast was performed to evaluate for pulmonary embolism, which was inconclusive. Consultants in Cardiology and Infectious Disease were consulted to participate in the care of the patient. Despite non-significant imaging, the patient was transferred to the intensive care unit and placed on a heparin drip for the management of a presumed pulmonary embolism (PE) with possible superimposed postpartum septicemia.

**Figure 2:**
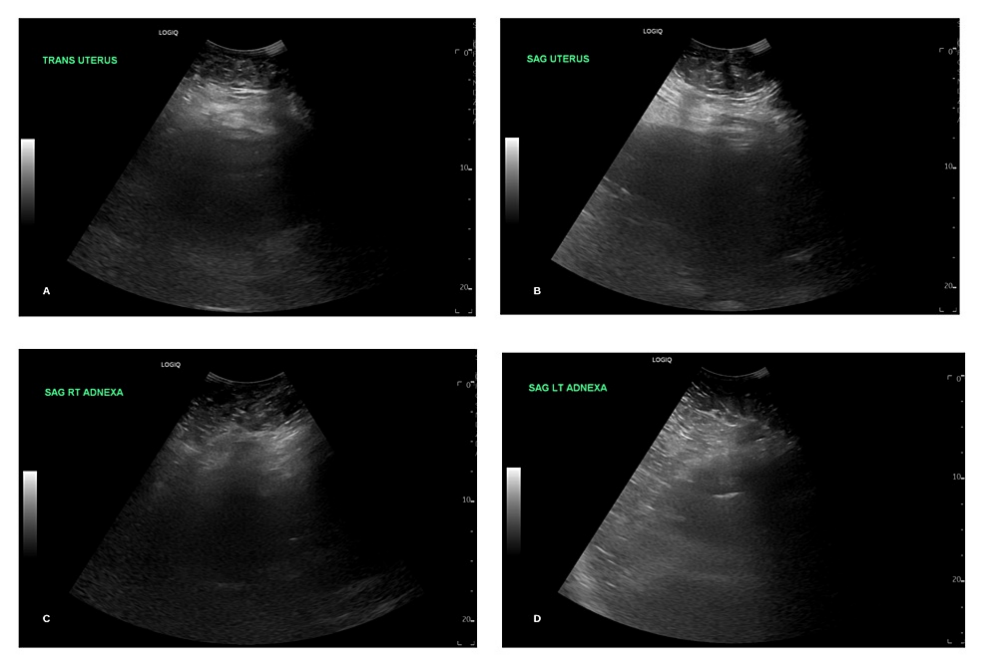
Pelvic ultrasound demonstrating negative findings; A: Transverse view of the normal uterus; B: Sagittal view of the normal uterus; C: Sagittal view of the normal right adnexa; D: Sagittal view of the normal left adnexa.

**Figure 3:**
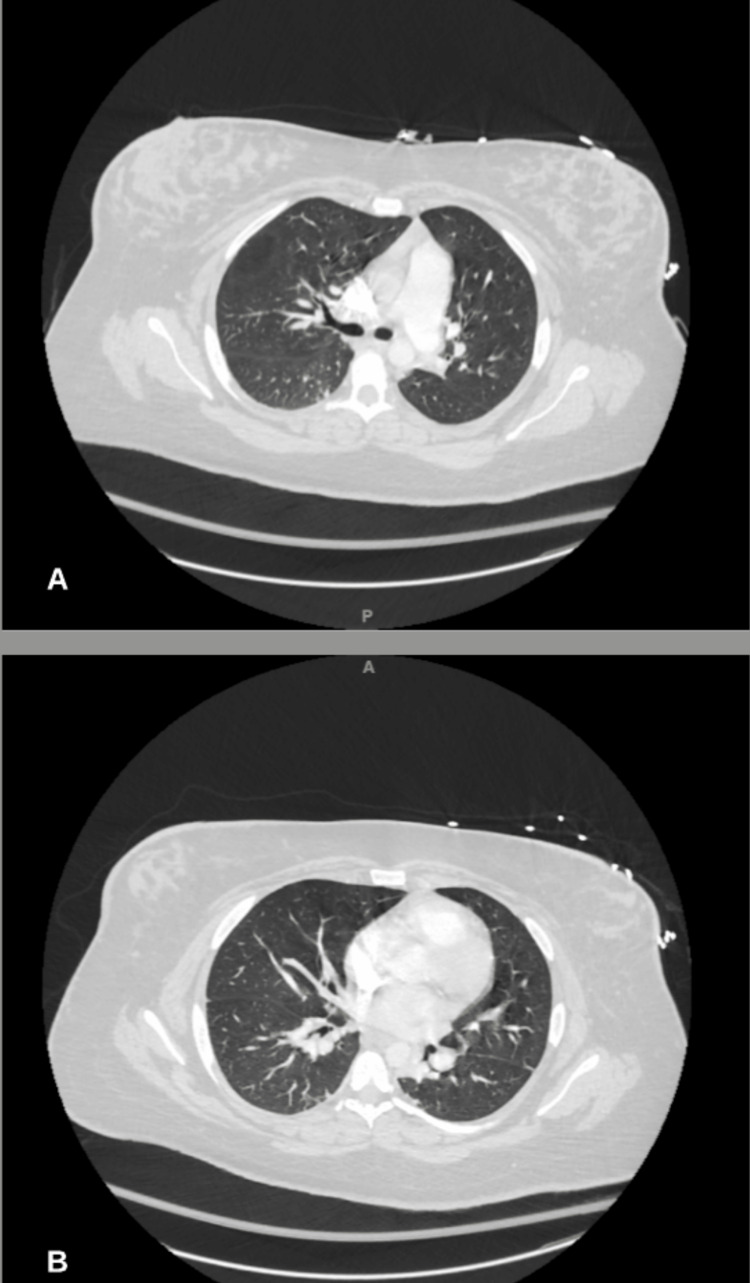
CTA unremarkable for pulmonary embolism or any other acute cardiopulmonary processes; A: Normal lower lung fields; B: Normal upper lung fields. CTA: computed tomography angiography.

**Figure 4:**
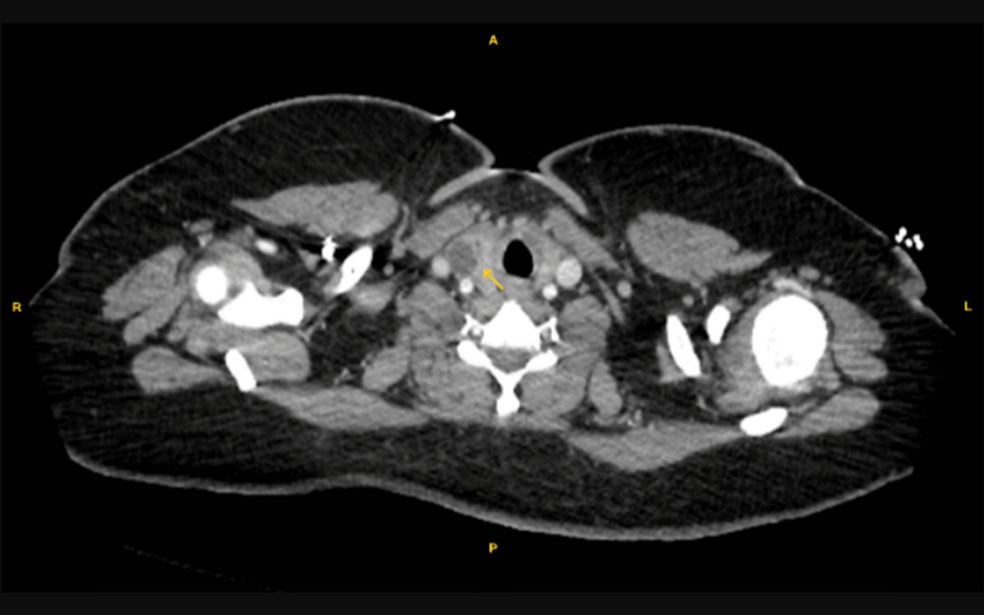
Incidental thyroid nodule noted on repeat CTA. CTA: computed tomography angiography. Yellow arrow: Thyroid nodule

Overnight between POD 1 and 2, the patient became diaphoretic and was persistently tachycardic, tachypneic, and hypercapnic with a blood pressure of 173/87. Her low-grade fever persisted with a temperature of 38.1°C. The WBC count had increased overnight to 21.7 x 10^9^/L. Blood cultures obtained were negative for any bacterial growth. The hemoglobin level decreased to 8.2 g/L, and the platelet count remained steady at 204 x 10^9^/L. The patient was placed on a 4L nasal cannula and had a blood oxygen saturation of 93% O2. Elevated blood pressures were attributed to the patient's pain and resolved after the administration of oxycodone as a component of a multimodal pain regimen. IV labetalol was on board as a pro re nata (PRN) medication, but administration was not required. The patient was given broad-spectrum coverage with clindamycin and cefepime to address the working diagnosis of postpartum septicemia. Additional imaging was ordered to evaluate for possible deep vein thrombosis (DVT). Bilateral lower extremity ultrasound was negative for any evidence of DVT, and pelvic ultrasound was similarly unremarkable. The patient remained in a similar condition on POD 3 with an additional elevation in WBC count to 23.6 x 10^9^/L. Abdominal and pelvic CT was ordered which were unrevealing of any pathology. A repeat CT angiography was not suggestive of PE, though the incidental appearance of a right lower lobe thyroid nodule measuring 1.9 cm was noted. At this juncture and in the absence of any pertinent imaging findings (Figures 2-4), the patient’s working diagnosis was changed to postpartum SPT.

With the new final diagnosis of postpartum SPT, the patient’s management was altered slightly over PODs 4 and 5. The heparin drip was discontinued and she was transitioned to therapeutic enoxaparin sodium. Additionally, initial broad-spectrum clindamycin and cefepime were changed to meropenem. Following this change, the patient’s WBC count decreased to 20.6 x 10^9^/L, though she remained febrile at 39.6°C, tachycardic, tachypneic, and hypertensive. She was considered stable enough to downgrade from the intensive care unit. The patient’s condition continued to improve with the addition of meropenem over the next several days, with her temperature normalizing to 36.6°C on POD 7, and the resolution of the tachypnea, tachycardia, and hypertension. Her WBC count continued to decrease to 16.1 x 10^9^/L. Final blood cultures obtained were negative for any growth after 5 days. She was transitioned from the nasal cannula back to room air with a blood oxygen saturation of 98%. It is interesting to note, that the patient remained non-toxic appearing throughout her hospital course, except 1 day of clamminess and diaphoresis on POD 2.

## Discussion

SPT is a rare condition with a strong correlation with the postpartum period. SPT was first described at the end of the 19th century by a 70-woman cohort researched by von Recklinghausen [1,3-4]. It is a challenging diagnosis, which is generally characterized by fever and antibiotic resistance. The condition affects nearly 1 in 3000 deliveries but is more frequent following cesarean sections, where it was found in 1 in 800 patients compared to 1 in 9000 vaginal deliveries [1-2]. A major route of pathophysiology is thought to involve Virchow’s triad (intravascular vessel wall damage, disturbance in blood flow within the vessels, and the presence of a hypercoagulable state) [5]. While SPT is not unique to the pregnancy and postpartum state, these time periods are an especially favorable host to these changes, considering the endothelial damage secondary to vaginal delivery or surgery, venous stasis secondary to decreased ovarian venous pressures, and ovarian venous dilation, and the generally increased hypercoagulability [1-2]. Other examples of patient populations that satisfy Virchow’s triad include those with atrial fibrillation, recent myocardial infarction, and malignancy [5]. While some cases have as few as one risk factor (generally the cesarean section), in this case, additional identifiable risk factors for hypercoagulation included the patient’s obesity and smoking history.

The disorder is sub-categorized into DSPT and ovarian vein thrombosis (OVT). The two types differ in clinical presentation but may occur concurrently and are thought to share the same underlying pathogenesis described above [2,6]. The right ovarian vein is involved in nearly 90% of OVT cases. This started to occur due to the increased length of the right vein compared to the left as well as left-to-rightward venous flow [4]. OVT patients are typically toxic-appearing and present with abdominal, flank, or back pain side of the thrombosed vein. In contrast, DSPT patients such as our case typically appear non-toxic, without associated abdominal pain, and present with antibiotic-resistant fever and leukocytosis beginning 3-5 days following delivery or surgery. These patients will also typically present with tachypnea and tachycardia, mimicking pulmonary embolism symptoms [1,4].

Due to the notable absence of significant laboratory and imaging findings, SPT has largely been considered a clinical diagnosis, and one of exclusion. Occasionally, CT or MRI may reveal thrombosis in the ovarian veins, however, imaging is often negative in the case of DSPT [3,7]. A recent study noted that blood cultures are negative upward of 97% of the time, as was the case with our patient [2]. However, if positive typically reveals *Streptococcus pyogenes* or *Escherichia coli *[2].

Treatment of SPT includes a mixture of broad-spectrum antibiotics and anticoagulation. Despite general suggestions, there are currently no set protocols or guidelines for the management of SPT, and data are mixed regarding the necessity of both aspects of treatment for the entirety of the episode [1,6]. Some sources suggest antibiotic treatment should continue for up to 72 hours following clinical improvement and resolution of the fever, while others note therapy should be maintained until discharge [2]. As blood cultures are generally negative, fever and leukocytosis tend not to respond to the initial broad-spectrum choice and antibiotics may need to be augmented. In the case of our patient, she did not initially respond to broad-spectrum antibiotics however leukocytosis resolved with the administration of meropenem. Anticoagulation should be continued for 48 hours following clinical improvement in patients without risk of hypercoagulation and for up to 2 months in patients without risk factors [2,8]. Currently, there is not enough data within the literature to endorse whether outcomes are improved with the administration of heparin vs enoxaparin. In our case, the patient was originally started on a heparin drip due to suspicion of pulmonary embolism but was transitioned to a therapeutic dose of enoxaparin for 2 weeks following admission. Complications of SPT include progression to septic pulmonary emboli or lung abscesses [4]. Our case is unique from many in the literature [2,4,6] in that, while pulmonary embolism was suspected, there was no confirmable evidence of septic emboli complicating the presentation.

## Conclusions

DSPT is frequently misdiagnosed and due to the life-threatening potential of the condition, clinical diagnosis should not be delayed. This case report aims to bring attention to this rare postpartum diagnosis. A high level of clinical suspicion for SPT and in particular, DSPT should be considered for postpartum patients presenting with persistent fever despite antibiotics around 4 hours following delivery and in the presence of negative imaging findings.
